# Central Nervous System Involvement by Waldenstrom Macroglobulinemia: A Case Report of the Bing–Neel Syndrome

**DOI:** 10.1155/2019/4075960

**Published:** 2019-03-14

**Authors:** Ananth Arjunan, Hema Rai

**Affiliations:** ^1^University of Pittsburgh Medical Center, Division of Hematology/Oncology, UPMC Cancer Pavilion, 5150 Centre Avenue, Room 463, Pittsburgh, PA 15232, USA; ^2^VA Pittsburgh Healthcare System, Division of Hematology/Oncology, University Drive C, 8th Floor, Pittsburgh, PA 15240, USA

## Abstract

Bing–Neel syndrome (BNS) is a rare complication of Waldenstrom macroglobulinemia (WM) defined by a lymphoplasmacytic infiltration of the central nervous system (CNS). Patients present with a range of neurologic symptoms of variable severity. Diagnosis requires a low threshold of suspicion and is considered in WM patients with unexplained neurologic symptoms. It can occur in the presence of quiescent serum markers of WM. Direct CNS tissue biopsy should be pursued if feasible and remains the gold standard for diagnosis. No standard of care treatment exists, but expert guidelines suggest intravenous chemotherapy in standard dose or high-dose regimens or use of oral ibrutinib. Consideration is also made for intravenous rituximab, intrathecal therapies, and autologous stem cell transplantation. Patient factors and tolerability should drive decisions regarding treatment choice in this arena, given a lack of data for standard frontline therapy.

## 1. Introduction

Waldenstrom macroglobulinemia (WM) is a B-cell lymphoproliferative disorder characterized by a monoclonal lymphoplasmacytic infiltrate in the bone marrow with production of circulating monoclonal IgM antibody. WHO classification designates WM as a lymphoplasmacytic lymphoma (LPL) [1]. WM is rare with less than 1500 cases diagnosed yearly in the United States [2]. Bone marrow evaluation shows an intertrabecular infiltrate of small lymphocytes, plasmacytoid lymphocytes, and plasma cells. Over 90% of patients exhibit the *MYD88*^L265P^ activating somatic mutation which leads to NF-*κ*B-dependent survival of LPL cells [3]. WM is incurable with median survival of 5 to 10 years. Clinical manifestations of WM result from either marrow infiltration by the LPL tumor cells or peripheral effects of the circulating monoclonal IgM. Marrow infiltration by the tumor cells leads to myelophthisic anemia and thrombocytopenia. Circulating IgM exerts deleterious effects through several mechanisms: (i) increased serum viscosity, (ii) deposition in end organs, and (iii) auto-antibody activity.

Neurologic manifestations are fairly common in WM. Hyperviscosity due to IgM-related erythrocyte agglutination can lead to stroke, confusion, headache, vision changes, and intracranial hemorrhage. Peripheral sensory neuropathy can result from IgM deposition in the peripheral nerves or from auto-antibody activity of IgM against myelin-associated glycoprotein in the peripheral nerve sheath [4]. An infrequent and poorly characterized neurologic disorder in WM patients, known as Bing–Neel syndrome, can be diagnosed when the dura, meninges, brain, or CSF are infiltrated by LPL tumor cells.

## 2. Case Presentation

A 60-year-old male presented with fatigue, sensory neuropathy, and lab findings of anemia and elevated albumin-globulin gap. Serum protein electrophoresis (SPEP) with immunofixation detected an IgM-kappa monoclonal protein quantitated at 3.9 g/dL. Free kappa-lambda light chain ratio was >390. Bone marrow evaluation revealed 70% marrow cellularity with kappa light chain restriction on staining. Bone marrow core biopsy evaluation showed 14% plasmacytes and plasmacytoid lymphocytes. A diagnosis of WM was made after serum IgM returned elevated at 12,500 mg/dL. The patient underwent plasmapheresis after developing hyperviscosity symptoms of blurred vision and headache. Subsequently, he received several lines of systemic therapy including rituximab-based and bortezomib-based treatment as well as ibrutinib. He was eventually hospitalized for fevers and altered mental status. Infectious work up, brain MRI, and routine CSF studies were unrevealing and he spontaneously improved. Several weeks later, he was hospitalized again with confusion and falls. Despite treatment of identified bacteremia and urinary tract infections, his mental status progressively worsened. Exam was notable for bilateral paratonia with sustained leg flexion, decorticate posture of right upper extremity, bilateral patellar hyperreflexia, and complete disorientation. MRI brain showed left cerebellar signal hyperintensity on T2 FLAIR sequence. Serum viscosity was within the normal range. EEG revealed no epileptiform activity. CSF had elevated protein and normal glucose levels, and all CSF infectious studies were negative. Cell counts showed only 2 WBC/mm^3^, and cytology and flow cytometry were limited by a paucity of cells. Paraneoplastic panels in serum and CSF were unrevealing. Immunofixation of CSF revealed a kappa-restricted IgM. Serum levels of IgM, free light chains, and beta-2 glycoprotein all were stable to improved.

Given the cerebellar enhancement noted on MRI and monoclonal IgM noted in the CSF, concern was raised for infiltration of CNS by his WM. Brain biopsy was offered but declined by the patient's family, given his overall clinical deterioration. A trial of empiric high-dose corticosteroid failed to yield improvement, and he was transitioned to hospice care.

## 3. Discussion

First described in 1936, involvement of the CNS by WM is eponymously titled as the Bing–Neel syndrome (BNS) [5]. BNS is relatively infrequent and is likely underrecognized. Fewer than 200 cases have been reported in our review of the literature. One review of 44 French patients with BNS showed median age at diagnosis of 63 years with 80% of patients of male sex. BNS was the first manifestation of WM in 36%. In the remaining patients, BNS was found at a median of 9 years after initial WM diagnosis with a median of 2 prior systemic therapies. Presenting symptoms were heterogeneous and included paresis, seizure, cord compression, confusion, and psychiatric derangements [6].

Direct biopsy of brain or meningeal tissue to assess for lymphoplasmacytic infiltrate is the gold standard and strongly recommended when it can be safely performed. CSF cytology and multidetector flow cytometry for immunophenotyping is advised. The culprit LPL cells always express CD19, CD20, CD52, and Ig-light chain and a majority express CD79b, CD11c, CD25, and surface IgM. CD5 and CD10 are usually negative [7]. Repeat CSF sampling increases diagnostic yield if initial cytology or flow cytometry are unrevealing. Quantitative PCR of CSF fluid to look for the *MYD88*^L265P^ mutation can be helpful [8]. Electrophoresis and immunofixation to identify monoclonal IgM in CSF can raise suspicion for BNS but are insufficient to confirm a diagnosis on their own. While monoclonal IgM in CSF may be from infiltrating LPL tumor cells, a disruption in the blood-brain barrier can allow peripheral IgM to enter the CNS. MRI imaging, preferably of the entire neuraxis, should be performed to assess for suspicious infiltrative lesions and rule out other causes. Findings on MRI may be described as diffuse infiltrative (leptomeningeal or periventricular white matter involvement) or tumoral (discrete lesions) but there are no established diagnostic radiologic criteria [9]. Brain MRI detects abnormalities in 78% of patients with this disorder; however, an absence of findings on MRI does not exempt the patient from an evaluation for BNS. Serum markers of WM may be stable or improved in patients with BNS and should not lower clinical suspicion for this entity.

Given the infrequency of BNS, there are no gold standard treatments and the opportunity for prospective trials is limited. A range of therapies have been utilized historically including intravenous and intrathecal chemotherapy, intravenous and intrathecal rituximab, radiation, and autologous stem cell transplantation. Oral ibrutinib has been shown in a recent case report to have CSF penetration with partial remission maintained in at 23 months of therapy [10]. One previously proposed treatment strategy established a dichotomy based on the degree of CNS involvement [11]. Group A was defined by direct CNS involvement and felt to benefit from chemotherapy and radiotherapy. Group B was defined by low CSF involvement (<5 cells/mm^3^) with symptoms driven by IgM deposition and thus plasmapheresis the mainstay of therapy. A concerted effort was recently made by the 8^th^ International Workshop on WM (IWWM-8) to produce diagnostic and therapeutic guidelines for BNS [12]. Several options proposed for symptomatic BNS include: (1) purine analogues, (2) bendamustine, (3) ibrutinib, or (4) high-dose methotrexate or cytarabine-based chemotherapy with potential additions of rituximab, intrathecal therapy, and autologous stem cell transplantation. Radiotherapy is effective in some case reports, but more data are needed to define its utility.

## 4. Conclusion

BNS is an infrequent complication of WM defined by CNS infiltration by lymphoplasmacytic cells. The median time from onset of symptoms to the diagnosis of BNS is 4 months [9]. The delay in achieving a diagnosis may be reflective of lack of uniformity in neurologic symptoms, lack of awareness of the disease state, and symptoms being incorrectly attributed to other WM-related neurologic disorders. BNS should be considered in WM patients with unexplained neurologic symptoms. Efforts should be made to obtain an MRI scan of the entire neuraxis, CSF sampling, and direct CNS tissue biopsy. Recent guidelines from IWWM-8 suggest use of a variety of cytotoxic chemotherapies or ibrutinib in the initial setting. Given a lack of clear standard of care therapy, emphasis should be placed on patient factors and tolerability in selection of a specific treatment regimen.
